# Comparison of Acetaminophen Degradation by Laccases Immobilized by Two Different Methods via a Continuous Flow Microreactor Process Scheme

**DOI:** 10.3390/membranes12030298

**Published:** 2022-03-05

**Authors:** Laura D. Sotelo, Diana C. Sotelo, Nancy Ornelas-Soto, Juan C. Cruz, Johann F. Osma

**Affiliations:** 1 CMUA, Department of Electrical and Electronics Engineering, School of Engineering, Universidad de los Andes, Cra. 1E No. 19A-40, Bogota 111711, Colombia; ld.sotelo@uniandes.edu.co (L.D.S.); dc.sotelo10@uniandes.edu.co (D.C.S.); 2 Department of Biological Sciences, Universidad de los Andes, Cra. 1E No. 19A-40, Bogota 111711, Colombia; 3 Laboratorio de Nanotecnología Ambiental, Escuela de Ingeniería y Ciencias, Tecnológico de Monterrey, N. L., Monterrey 64849, Mexico; ornel@tec.mx; 4 Department of Biomedical Engineering, School of Engineering, Universidad de los Andes, Cra. 1E No. 19A-40, Bogota 111711, Colombia; jc.cruz@uniandes.edu.co

**Keywords:** microreactors, laccase immobilization, acetaminophen, micropollutant, wastewater treatment

## Abstract

The presence of micropollutants in wastewater is one of the most significant environmental challenges. Particularly, pollutants such as pharmaceutical residues present high stability and resistance to conventional physicochemical and biological degradation processes. Thus, we aimed at immobilizing a laccase enzyme by two different methods: the first one was based on producing alginate-laccase microcapsules through a droplet-based microfluidic system; the second one was based on covalent binding of the laccase molecules on aluminum oxide (Al_2_O_3_) pellets. Immobilization efficiencies approached 92.18% and 98.22%, respectively. Laccase immobilized by the two different methods were packed into continuous flow microreactors to evaluate the degradation efficiency of acetaminophen present in artificial wastewater. After cyclic operation, enzyme losses were found to be up to 75 µg/mL and 66 µg/mL per operation cycle, with a maximum acetaminophen removal of 72% and 15% and a retention time of 30 min, for the laccase-alginate microcapsules and laccase-Al_2_O_3_ pellets, respectively. The superior catalytic performance of laccase-alginate microcapsules was attributed to their higher porosity, which enhances retention and, consequently, increased the chances for more substrate–enzyme interactions. Finally, phytotoxicity of the treated water was lower than that of the untreated wastewater, especially when using laccase immobilized in alginate microcapsules. Future work will be dedicated to elucidating the routes for scaling-up and optimizing the process to assure profitability.

## 1. Introduction

Wastewater may be defined as a byproduct of domestic, industrial, commercial, or agricultural activities. Most human activities that involve water produce wastewater and, as the overall demand for water will continue to grow in the years to come, the volume of wastewater produced, and its overall pollution load, will also continue to increase worldwide [1,2]. In this regard, wastewater treatment has become an important worldwide priority over the past decade. If wastewater is not properly treated, it may negatively impact the environment and human health, thus, resulting in a costly problem to most governments and environmental agencies. At a global scale, around 80% of wastewater produced is discharged into the environment untreated, causing widespread water pollution. As a result, around the world, water contaminated by bacteria, nitrates, phosphates, and solvents is discharged into rivers and lakes, ending up polluting the oceans, with negative consequences for several aquatic ecological niches and even public health [3]. 

Wastewater characteristics vary depending on the source and type of contaminants, which, in turn, can be categorized into physical, chemical, or biological. However, most successful treatment approaches are principally based on the degradation of micropollutants [4,5], which are compounds present in the range of nanograms to micrograms per liter, and that exhibit high toxicity, persistence and/or a marked tendency to accumulate in the tissues of living organisms [6]. Micropollutants can be categorized according to their use into pharmaceuticals, personal care products, household chemicals, and industrial agents.

One of the major challenges with micropollutants is that conventional wastewater treatment technologies such as activated sludge fail to eliminate them completely or even to reduce their concentrations to permissible levels [6]. Enzymatic treatment, however, has emerged as a viable alternative for biotransformation in different areas [7], particularly in the case of organic micropollutants. This is related to enzymes’ lower susceptibility to inhibition by substances that are usually toxic to living organisms. Moreover, they show remarkable stereo- and regio- selectivity and may be effectively used over a broad concentration range, even in diluted solutions [8]. Moreover, they enable processes with reduced consumption of reagents, water, and energy, while reducing the undesirable byproducts [9]. Consequently, industrial processes based on enzymatic technologies are often the choice when it comes to assuring lower environmental impact and more sustainable processing routes [10,11].

A successful example of such approaches is the numerous processes enabled by enzymes from the oxidoreductase family, which can catalyze oxidation and reduction reactions very efficiently [6,12]. In such schemes, these enzymes transfer electrons from one molecule (the reductant) to another (the oxidant). This capability has been exploited in the development of an ample variety of processes where redox reactions are ubiquitous e.g., dye degradation, food industry and wastewater treatment [13,14,15]. In particular, fungi oxidoreductase enzymes, including the white-rot laccase, lignin peroxidase (LiP), and manganese peroxidase (MnP), have been synergistically employed to oxidatively and non-selectively cleave the recalcitrant polyphenol structures of lignin during the decomposition of wood [16]. These enzymes have also been employed to degrade several organic micropollutants, including pharmacological compounds such as Diclofenac, Trimethoprim, Carbamazepine, and Sulfamethoxazole, with remarkable efficiencies of 100%, 95%, 85%, and 56%, respectively [9,12,17]. The foregoing is of great importance, since a large portion of most of the pharmaceutical compounds is excreted unmetabolized in urine and feces upon consumption [18]. Among these, 58–68% of acetaminophen is excreted from the body when ingested in therapeutic dosage, making it one of the most commonly found pharmaceutical contaminants in wastewater [18,19]. Although its concentrations are usually at the ppt to ppm levels, it can transform into various intermediates; its degradation products make it a challenging contaminant for most wastewater treatment technologies [19].

The degradation reactions catalyzed by oxidoreductases generally follow the production of free radicals driven by the redox potential difference between the enzyme active site and the substrate. This results in substrate oxidation and in the generation of polymerization prone phenoxy free radicals [4]. Through this mechanism, Laccase, LiP and MnP have been previously shown to decompose phenolic and aromatic compounds, which usually show relatively low redox potential [4,6,20]. Furthermore, their applicability has been expanded to non-phenolic or non-aromatic substrates, including dyes, active residues from pharmaceuticals, pesticides, surfactants, and hormones [4,21].

One of the major challenges of incorporating enzymes into bioremediation processes is their limited ability to tolerate harsh pH, ionic force, temperature, and environmental conditions [22]. Under such conditions, enzymes tend to exhibit a significant loss of activity mainly due to substantial conformational changes that result in active site disruption [23]. A successful strategy to overcome this major hurdle is the encapsulation or immobilization of the enzyme molecules on different types of materials, including natural and synthetic polymers, fabrics, ceramics, metals and, more recently, nanostructured materials [24]. Immobilization or encapsulation can be achieved either by physical adsorption or by covalent bonding. Nanostructured materials such as titania, alumina, silica and magnetite offer rather simplified immobilization routes, high chemical and physical stability, ease of handling, low costs, and relatively high yields [10,11,25,26,27]. In contrast, microencapsulation has been employed extensively as an efficient route to protect or isolate substances from harmful environments [28], hence, serving as a viable solution for oxidative degradation. Moreover, the encapsulated substances can be released in a controlled manner, without significantly altering their properties and specific functionalities [29,30]. Microencapsulation may take place either by physical or chemical methods; within this category ionic gelation is, perhaps, one of the favorite ones because of its low cost, the inertness of the involved materials, the ease of implementation, and scalability [27,28].

One of the most widely used polymers for microencapsulation is alginate, which undergoes a mild gelation process when in contact with divalent ions, such as Ca^2+^, Mg^2+^ and Cu^2+^, by forming a stable polymer network [29]. Nevertheless, some of the major challenges of conventional microcapsule manufacture methods include the precise control of morphology, size distribution and porosity of the formed capsules. This poses a major challenge to assure ease of scalability and the eventual economic success of the processes. Alternatively, recent research in our group, carried out by Campaña, A. et al. [29], has suggested that droplet-based microfluidic systems offer a more controlled route for the assembly of the microcapsules and for the chance to reduce the number of reagents required for manufacturing, thus, providing an inexpensive manufacture process. This adds up to the low-cost manufacturing approach developed by our research group over the past few years, where microsystems are cut by laser-assisted techniques and easily assembled with the aid of inexpensive adhesives and homemade press systems. Moreover, the microchannels are engraved on polymethyl methacrylate (PMMA) substrates also accessible locally at very low costs. Besides these attractive features, compared with conventional reaction systems, microreactors offer low energy consumption, high reaction rates and yields, ease of scalability and, most importantly, the possibility of incorporating process control [31].

Here, we aimed at comparing the catalytic performance of laccase encapsulated into monodisperse alginate-based microcapsules obtained through a droplet-based microfluidic system and laccase immobilized via covalent binding on aluminum oxide pellets. The performance was assessed in continuous flow microreactors, manufactured by laser-cutting techniques, by measuring acetaminophen degradation in model wastewaters. Moreover, we examined the reusability of the two laccase immobilization methods and the toxicity of the treated and untreated wastewater using a phytotoxicity model based on the growth of sawgrass seeds. We hypothesized that the laccase immobilization method used for the wastewater treatment would have a significant impact on the acetaminophen degradation and enzyme loss. 

## 2. Materials and Methods

### 2.1. Reagents and Materials

All materials were used as received and with no further purification. 2,2′-azino-bis (3-ethylbenzothiazoline-6-sulfonic acid) diammonium salt (ABTS), copper (II) sulfate (CuSO4), were purchased from Sigma Aldrich (St. Louis, MO, USA). Γ-Aminopropyl-triethoxysilane (APTES) (98%), glutaraldehyde (25%), hydrochloric acid (HCl), sodium hydroxide (NaOH), potassium dihydrogen phosphate (KH_2_PO4) and potassium hydrogen phosphate (K_2_HPO4) were purchased from Merck, (Darmstadt, Germany). 91% (*w*/*v*) Acetaminophen was purchased from Tecnoquimicas (Bogota, Colombia). Alginic Acid Sodium Salt BioChemica (Sodium alginate) (90.8%) was obtained from PanReac AppliChem (Barcelona, Spain). Bradford Protein Assay kit was purchased from Bio-Rad (Hercules, CA, USA). Commercial soybean vegetable oil, solvent-based acrylic glue, methylene chloride and polymethylmethacrylate (PMMA) sheets of 2 mm thickness were obtained from local distributors (Bogota, Colombia). 1100 µm diameter aluminum oxide (Al_2_O_3_) pellets were purchased from International XIETA S.L. (Barcelona, Spain). Laccase from *P. Sanguineus* CS43 was obtained as described elsewhere [32].

### 2.2. Laccase Immobilization on Silanized Al_2_O_3_ Pellets

Laccase immobilization was carried out on silanized Al_2_O_3_ pellets using glutaraldehyde as crosslinker. The process started by suspending 2540 mg of Al_2_O_3_ pellets in 40 mL of milli-Q water. Next, 800 μL of 0.5 M NaOH was added and mixed vigorously for 20 min. Then, 500 µL of 2% (*v*/*v*) APTES was added, followed by mixing at 25 °C and 400 RPM for 30 min. Once pellets were silanized, 500 µL of 2% (*v*/*v*) glutaraldehyde was added and left under mechanical agitation at 300 RPM and room temperature for 60 min. Pellets were washed thoroughly 3 times with milli-Q water to remove the excess of unbonded glutaraldehyde and were then resuspended in 5 mL of milli-Q water. Next, 32 µL of 53,500 U/L Laccase was added and vortexed at room temperature for 3 min. Then, the functionalized pellets were left overnight under static conditions at room temperature. Finally, pellets were washed thoroughly 2 times with milli-Q water, to remove the excess of unbonded laccase, and were stored at 4 °C until further use.

### 2.3. Laccase Encapsulation into Alginate Microcapsules via Microfluidics

The design and manufacture of the microfluidic device was carried out following the procedure described by Campaña, A. et al. [29]. As described by the authors, a flow-focusing approach was selected to manufacture the polymeric capsules, due to the superior control over the size of the microcapsules. Fabrication of the microsystems was carried out using laser-cutting techniques and Polymethylmethacrylate (PMMA) sheets as substrate to form the microchannels required for interaction of laccase molecules and alginate. Prior to running the system, water was pumped through the microchannels to assure proper sealing. A sodium alginate solution 1% (*w*/*v*) was prepared for the microcapsules generation; the flow rates were set to 80 mL/h (continuous phase flow, Q_c_) and 0.7 mL/h (discrete phase flow, Q_d_). Flow rates were chosen to obtain a size distribution of around 1100 µm for the microcapsules. 

Microcapsules were produced using the manufactured microfluidic device, infusing soybean vegetable oil as continuous phase (Q_c_) and the sodium alginate solution as discrete phase (Q_d_). In the same way, a 3% (*w*/*v*) Copper sulphate solution (CuSO_4_) was used for microcapsule gelation at room temperature; the size distribution was estimated using an in-house application developed in MathWorks MATLAB^®^ (MathWorks, Natick, MA, USA), with the aid of a 1000X Digital USB Microscope, as described by Campaña, A. et al. [29].

To obtain a final solution with a 1:5 ratio, a mixture of 200 µL of laccase solution (53,500 U/L) and 800 µL of 1% (*w*/*v*) sodium alginate solution was used as the discrete flow; the same parameters described above were set for the laccase encapsulation. Once the microcapsules were formed inside the device, they were cross-linked in the CuSO_4_ solution for 8 h and, subsequently, stored at 4 °C until further use.

### 2.4. Laccase Encapsulation/Immobilization Efficiencies

Encapsulation/immobilization efficiency was determined indirectly by Equation (1). This method relies on the mass difference between free and residual laccase [33]. In this sense, the immobilization ratio and activity recovery can be calculated based on the total units of enzyme present either in the immobilized system or the supernatant after immobilization. Thus, according to Equation (1), *A* refers to the initial enzyme concentration multiplied by the used volume; *B* refers to the enzyme concentration multiplied by the volume of the supernatant solution after immobilization. Laccase concentration was measured spectrophotometrically using the Bradford protein assay.

(1) $$\mathrm{Efficiency}\;(\%)=\frac{\left(A-B\right)}{A}\times 100$$

### 2.5. Quantification of Laccase

The amounts of free laccase, as well as laccase loss inside the microreactor after wastewater treatment, were qualitatively assessed with the aid of the Bradford protein assay. Laccase activity was measured by the ABTS assay following the approach developed by Niku-Paavola et al. [34]. Enzymatic activity was measured in a Genesis 10S spectrophotometer (Thermo Fisher Scientific, Waltham, MA, USA) for 1 min at 436 nm. One activity unit was defined as the amount of laccase needed to oxidize 1 µmol of ABTS per minute under the assay conditions. Laccase activity was expressed in terms of units per liter (U/L). In the case of continuous-flow operation of the microreactor in the presence of acetaminophen-containing wastewater, 200 µL was collected at the outlet of each microreactor and was analyzed after each operation cycle. 

### 2.6. SEM Characterization

Alginate-based microcapsules and Al_2_O_3_ pellets functionalized with laccase enzymes were observed with a JEOL Lyra 3 (TESCAN, Brno, Czech Republic) focused ion beam-scanning electron microscope (FIB-SEM, TESCAN instrument, Brno, Czech Republic). Observation of microcapsules and Al_2_O_3_ pellets was carried out under cooling conditions in a cooling stage system to avoid any disruption on the morphology caused by the vacuum conditions required. Furthermore, an energy-dispersive X-ray spectroscopy (EDS) analysis was carried out to identify possible traces of copper in the microcapsules, carbon and silicon in the pellets, and nitrogen in both.

### 2.7. Design of the Continuous Flow Microreactor for Acetaminophen Treatment

**Figure 1:**
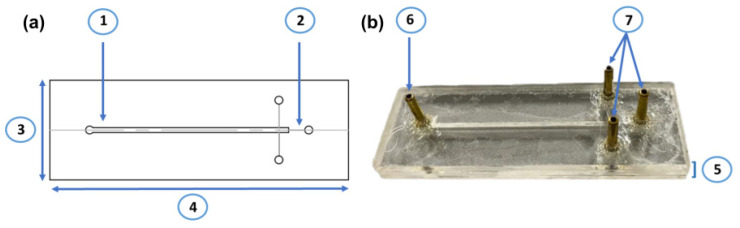
Microreactor design. (**a**) Blueprint of the microreactor, ① main microchannel, ② outlet microchannels, ③ Width of 2.5 cm and ④ Length of 7.5 mm. (**b**) Photograph of a microreactor, ⑤ thickness of 6 mm, ⑥ inlet connector and ⑦ outlet connectors.

The design of microreactors was carried out using AutoCAD^®^, as shown in Figure 1. The microreactor design was based on two main principles: letting the water flow from one inlet to multiple outlets, and the retention of microcapsules or pellets with immobilized laccase. Therefore, the microreactor consisted of four main parts, the main microchannel, where the microcapsules or pellets are confined (Figure 1 ①), the three outlet microchannels (Figure 1 ②), the inlet connector (Figure 1 ⑥) and the outlet connectors (Figure 1 ⑦). The main microchannel had a width of 1.2 mm and a depth of 2 mm to store the microcapsules and pellets, while the three outlet microchannels had a width of 0.4 mm and a depth of 2 mm to avoid significant losses of microcapsules or pellets. Outlet microchannels were designed (a third of the main microchannel in width) to obtain a proportional flow distribution and, thus, to attain proportional pressure and velocity conditions in each of the water outlet connectors. The width, length and thickness of the microreactor corresponded to 2.5 cm (1”) (Figure 1 ③), 7.5 cm (3”) (Figure 1 ④), and 6 mm (0.236”) (Figure 1 ⑤, respectively. The fabrication method and materials of the microreactor were the same described previously by Campaña, A. et al. [29] for microcapsule generation.

### 2.8. Acetaminophen Treatment in Microreactors with Immobilized Laccase

**Figure 2:**
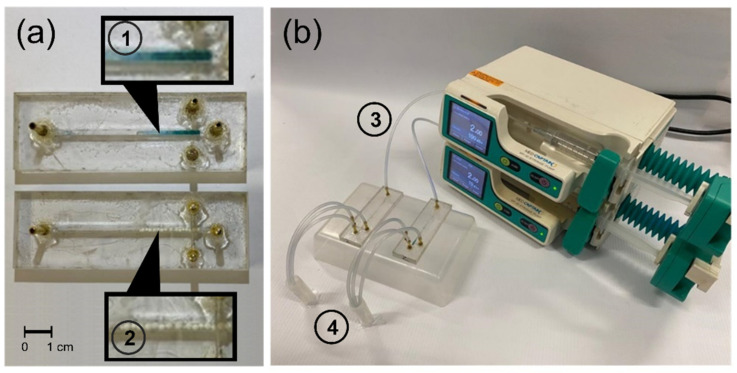
Experimental setup for wastewater treatment. (**a**) Microreactors for acetaminophen treatment filled with ① Laccase-alginate microcapsules and ② Laccase-Al_2_O_3_ pellets up to 2 cm of the main microchannel. (**b**) Assembly carried out for wastewater treatment. ③ Wastewater samples were pumped through the inlet and ④ treated water was collected at the end of each cycle.

**Figure 3:**
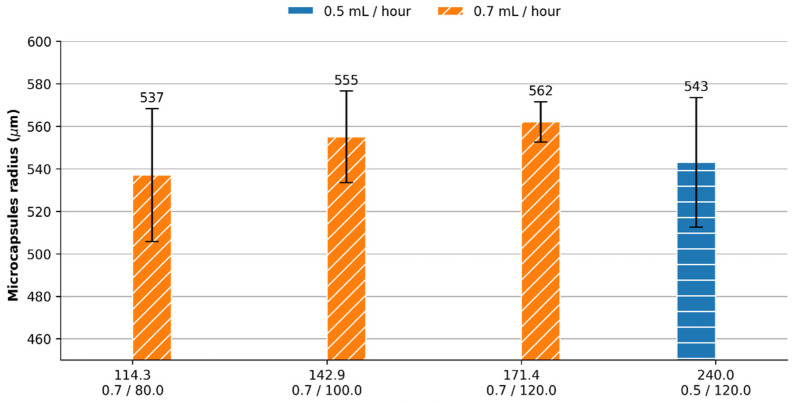
Mean radius of microcapsules upon Q_c_/Q_d_ ratio. Error bars correspond to the standard deviation.

To assess the catalytic efficiency of the immobilized laccase in the degradation of acetaminophen, a model artificial wastewater solution was prepared by diluting acetaminophen in milli-Q water at a concentration of 18 mg/L. For the degradation assay, 2 cm length (out of the 5 cm) of the main microchannel of each microreactor was filled with microcapsules or pellets with immobilized laccase (Figure 2a, ① and ②). A total of 3 mL of the acetaminophen-containing wastewater was pumped, at a flow rate of 2 mL/h through the continuous-flow microreactors (Figure 2b, ③). Tests were conducted in triplicate.

**Figure 4:**
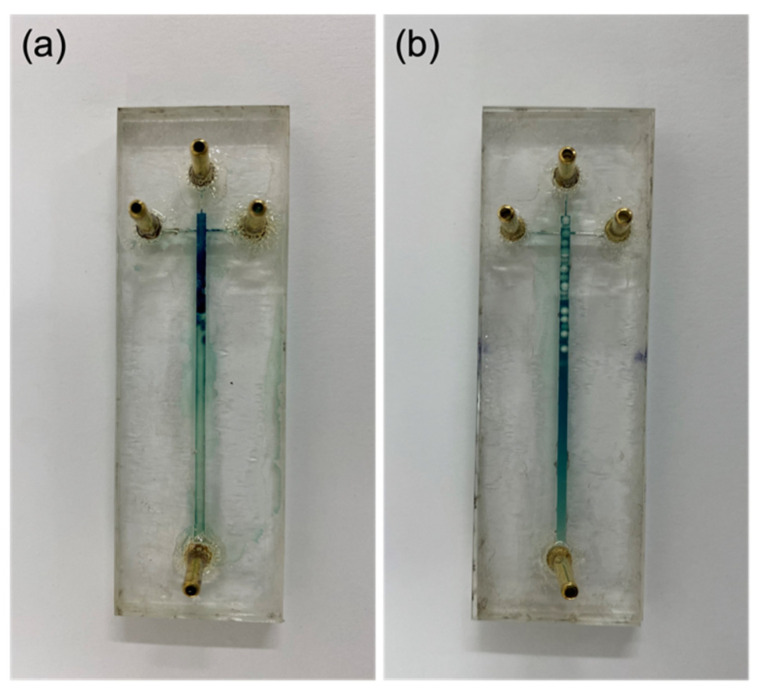
Immobilized laccase activity test. Microreactors with (**a**) Laccase-alginate microcapsules and (**b**) Laccase-Al_2_O_3_ pellets showing catalytic activity in a ABTS assay.

Each operation cycle was completed after pumping 1 mL of acetaminophen-containing wastewater. The treated water was collected and placed in a measurement cell for further analysis (Figure 2b, ④). Acetaminophen was quantified spectrophotometrically at 244 nm [12]. Each microreactor filled with microcapsules or pellets was operated for three cycles. Treatments were carried out at different pH values, 3.0, 4.5 and 6.0, using potassium phosphate buffers.

### 2.9. Phytotoxicity Test

Three sawgrass seeds (Fercon, Cali, Colombia) were placed on round filter paper in a Petri dish and exposed to untreated or treated wastewater samples obtained from each microreactor test. In this sense, three wastewater samples corresponding to pH values of 3.0, 4.5 and 6.0 were tested. Milli-Q water was used as the positive control, while untreated acetaminophen-containing wastewater (18 mg/L) was used as the negative one. All tested samples were diluted 1:1 in milli-Q water to cover properly the filter paper with the sawgrass seeds. Lastly, seeds were grown for five days under static conditions and eight-hour light cycles. Phytotoxicity was determined by counting the germinated seeds and by measuring the stem and roots lengths. Tests were conducted in duplicate, each one by considering three seeds.

### 2.10. Statistical Analysis

Data was analyzed using single factor ANOVA for each experiment. Tukey tests were carried out as the post hoc test to determine the significance in the differences between studied groups. All analyses were done using an alpha value of 0.05.

## 3. Results

### 3.1. Laccase Encapsulation into Alginate Microcapsules via Microfluidics

The goal of this experimentation stage was to determine the optimal continuous (Q_c_) and discrete flow rates (Q_d_) to produce laccase-alginate microcapsules with a homogeneous diameter distribution around 1100 µm using the flow-focusing microfluidic device for microcapsules generation [29]. The diameter was selected to match as close as possible that of the Al_2_O_3_ pellets.

According to Campaña, A. et al. [29], variations of input flow rates, reagents concentration and channel diameter might have a significant impact on microcapsules size distribution for flow-focusing microfluidic systems. Therefore, here, we tested four different combinations of Q_c_ and Q_d_ flow rates. The mean ratio of the microcapsules produced for a Q_d_ value of 0.7 mL/h was 557 ± 8 µm, while that of the ones produced with a Q_d_ value of 0.5 mL/h was 543 ± 15 µm. As reported by the authors, changes in Q_d_ flow rate showed no significant impact on the capsule size and the size distribution of the capsules decreased with an increase of the Q_c_ flow rate. 

After testing different Q_d_ flow rates, it was set to 0.7 mL/h, while the Q_c_ flow rate was varied from 120 mL/h to 80 mL/h every 20 mL/h, where it was found that this variation results in an increase in radius of about 100 microns, as shown in Figure 3. Accordingly, the optimal flow rate ratio to obtain a size distribution close to 550 µm of radius (1100 µm of diameter) was for a (Q_c_/Q_d_) of (80 mL/h)/(0.7 mL/h) and, thus, it was selected for further experimentation. This size distribution avoids a significant loss of microcapsules through the outlet of the microreactor and is similar to that of the Al_2_O_3_ pellets. Laccase-alginate microcapsule generation was carried out using this flow rate and led to an average diameter of 1108 ± 31.28 µm.

### 3.2. Laccase Immobilization Efficiencies

The extent of encapsulation efficiency was attained by measuring the concentration of the immobilized enzyme and the supernatant after encapsulation and functionalization processes by following Equation (1). The encapsulation efficiency for laccase-alginate microcapsules 92.18%, while the functionalization (Al_2_O_3_ pellets) efficiency was 98.22%. Prior to each degradation test, the activity of the immobilized laccase was confirmed by the oxidation of ABTS, as shown in Figure 4, where the dark green color corresponds to oxidized ABTS. 

### 3.3. Microscopy Characterization of Immobilized Pellets and Microcapsules

**Figure 5:**
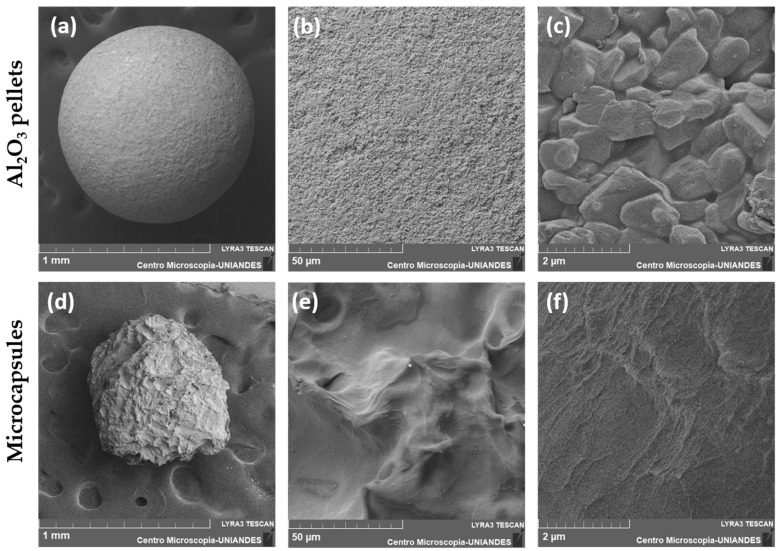
SEM micrographs of laccase-Al_2_O_3_ pellets and laccase-alginate microcapsules. Observation was carried out with a magnification of (**a**,**d**) 150×, (**b**,**e**) 2000× and (**c**,**f**) 40,000× after 7 days of fabrication.

SEM micrographs of laccase-Al_2_O_3_ pellets demonstrated a spheric, compact and granular structure (Figure 5a–c), while Laccase-alginate microcapsules (Figure 5d–f) exhibited a typical rounded-shape morphology and some surface protrusions. Imaging was carried out 7 days after laccase immobilization. These results were similar to those reported by Campaña, A. et al. [29] for the microcapsules after 20 days of fabrication, which confirm that microcapsules are stable for about one to two weeks. Although microcapsules suffered some mechanical stress during observation, both, Al_2_O_3_ pellets and microcapsules, had roughly the same size, which is an important feature for the correct functionality of the acetaminophen-treating microreactor.

**Figure 6:**
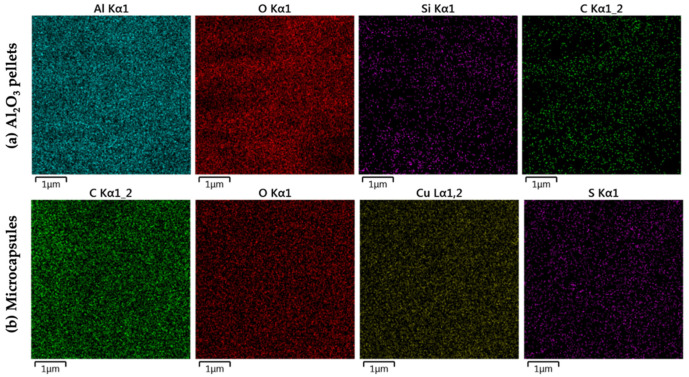
EDS analysis. Distribution of elements for (**a**) where aluminum, Oxygen, silicon, and carbon channels are shown; and (**b**) where carbon, oxygen, sopper and sulfur channels are shown.

EDS analysis of laccase-Al_2_O_3_ pellets (Figure 6a) showed the presence of aluminum and oxygen, which are highly abundant on Al_2_O_3_ pellets. silicon and carbon were found to be uniformly distributed on the surface of the immobilized laccase pellets. Silicon evidenced the presence of the silane linker used to bind the laccase, while the carbon can be attributed to both linkers and enzymes. EDS analysis of laccase-alginate microcapsules (Figure 6b) showed uniform distribution of carbon, oxygen, copper and sulfur that can be attributed to the alginate (i.e., carbon and oxygen), the salt (i.e., copper and sulfur), and the enzyme (i.e., carbon, oxygen, copper and sulfur). 

Lastly, the distribution map of elements that make up the sample is shown in Figure 6, where it is observed that there is a homogeneous distribution of elements on the surface. No areas with an increase of any specific element were detected; on the contrary, they were all distributed across the pellets and microcapsules surface.

### 3.4. Acetaminophen Treatment in Microreactors Packed with Immobilized Laccase Preparations

Acetaminophen degradation was studied using different microreactors. Half of them with Laccase-alginate microcapsules and the other half with Laccase-Al_2_O_3_ pellets, each of which had media adjusted to different pH values. Three samples of the effluent of each of the microreactors were collected at 1800 s, 3600 s, and 5400 s after starting each operation cycle. 

**Figure 7:**
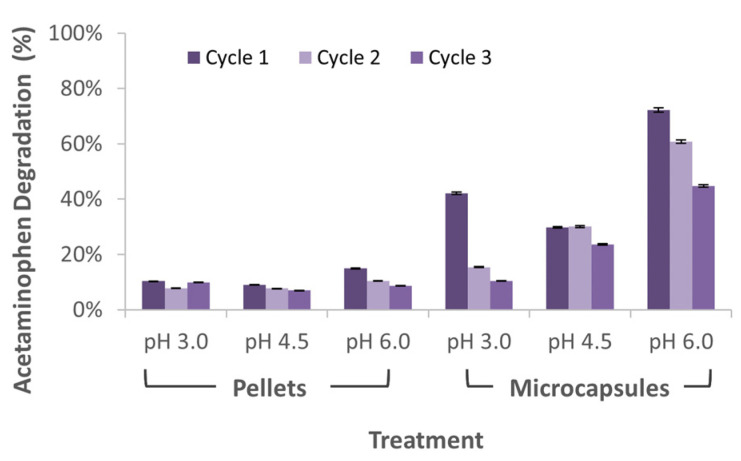
Relative concentration of acetaminophen in the eluded samples of microreactors. Each cycle was set to a different pH for the treatment of acetaminophen. Relative concentration was calculated based on Lambert-Beer law for both pellets and microcapsules.

Since the degradation experiments were conducted under a continuous flow regime of the model wastewater, results showed that the acetaminophen was likely adsorbed on the microcapsules and Al_2_O_3_ pellets. Figure 7 shows that, for a pH of 6.0, a greater concentration of acetaminophen was degraded, reaching a maximum removal percentage of 72% and 15% for the microcapsules and pellets, respectively. The superior removal capacity of the laccase-alginate microcapsules can be largely attributed to their porosity, which favors a larger number of acetaminophen molecules to come in close contact with the active sites of the laccase molecules for longer periods of time [29]. This was not the case for the laccase-Al_2_O_3_ pellets, possibly due to lower adsorptive properties, since they behaved more like a solid barrier that increased the wastewater velocity and showed much less affinity for the acetaminophen molecules, most likely due to the low sorption properties compared to that of alginate [35,36]. This combined effect leads to a much shorter contact time and, consequently, a reduced capacity for biotransformation.

As shown in Figure 7, when the wastewater effluent was set to pH 6.0, acetaminophen degradation was the highest, similar to the results reported by Chen et al. 2012 [37], where it was observed that the acetaminophen oxidation peak was higher at a pH value of 6.0 and dropped under acidic conditions. Additionally, a consecutive decrease in the degradation capacity was observed for cycles 2 and 3. This decrement in acetaminophen degradation can be attributed to enzymatic inhibition processes possibly induced by mechanical damage from successive reactions or to enzyme leakage. In the case of the laccase-Al_2_O_3_ pellets, the greatest enzyme inhibition approached 40% for the experiment at pH 6.0, while, for the laccase-alginate microcapsules, it was 80% for the pH 3.0, from the first to the third cycle. According to the ANOVA analysis, the use of laccase-alginate microcapsules had a significant impact on the acetaminophen degradation (F(5,12) = 13.41 and *p* = 0.000145). Tukey’s test showed that the treatment with microcapsules at pH 6.0 was significantly different than the other treatments, including those using microcapsules at other pH values.

### 3.5. Released and Laccase Losses during Wastewater Treatment

**Figure 8:**
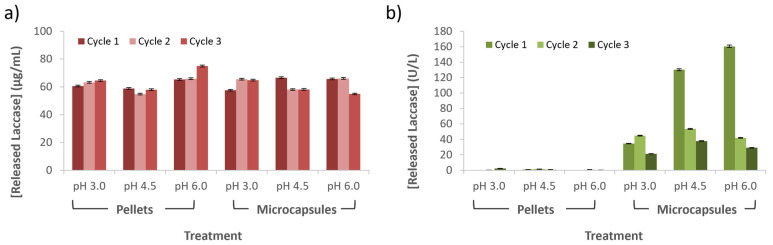
Laccase losses during acetaminophen treatment using a model artificial wastewater. (**a**) Laccase losses measured in terms of concentration and (**b**) enzymatic activity.

Immobilization and encapsulation efficiencies were calculated based on the mass difference between the initial free laccase used and the residual laccase recovered in the supernatants after each treatment process by following Equation (1). Laccase concentration was measured spectrophotometrically in triplicate using a commercial Bradford protein assay. As shown in Figure 8a, laccase losses, measured in terms of concentration, were about constant between cycles. According to the ANOVA analysis, the immobilization method had little impact in the laccase losses in terms of concentration (F(5,12) = 2.061 and *p* = 0.1413). Nevertheless, much more marked enzyme losses were observed for cycle 2 in microreactors operating with laccase-alginate microcapsules compared with experiments carried out with laccase-Al_2_O_3_ pellets (Figure 8b). This can be explained by the covalent immobilization route employed to form the laccase-Al_2_O_3_ pellets. According to the ANOVA analysis, the immobilization method had a significant impact on the cumulative laccase losses in terms of activity units (F(5,12) = 771.60 and *p* = 1.29 × 10^−14^). Tukey’s test showed that the laccase losses in terms of activity of laccase-alginate microcapsules were significantly different than those relating to Al_2_O_3_ pellets. In the same way, laccase cumulative losses in terms of activity of laccase-alginate microcapsules at pH 4.5 and 6.0 presented significant differences compared to the losses of the same immobilization method at pH 3.0.

**Figure 9:**
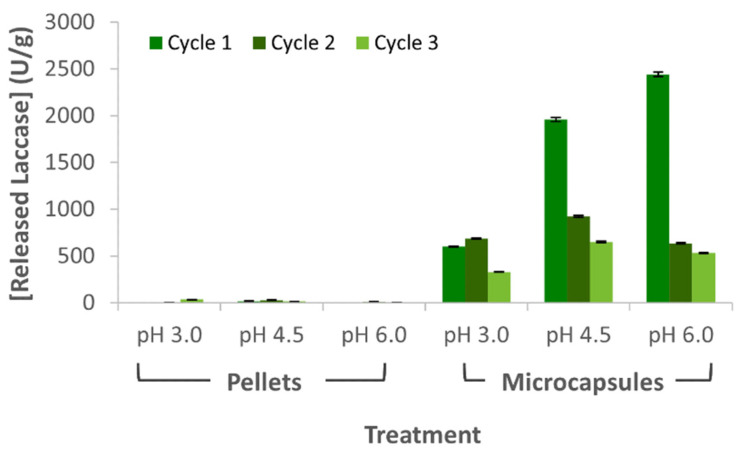
Ratio of laccase loss in terms of activity per amount of laccase. Measurements were carried out for water samples collected after treatment.

Figure 9 shows that, despite the considerable amounts of laccase molecules lost after cycle 2 from the microcapsules, their enzymatic activity was well maintained, except for the experiment at pH 3.0. However, as cycles progressed, the enzymatic activity was gradually lost, which could explain the decrease in acetaminophen degradation. Despite that the amount of enzyme molecules lost from pellets was similar to that lost from capsules, the achieved activity was well below that of microcapsules, which provides further evidence that the immobilization mechanism, based on crosslinking, might have had a detrimental impact on the native structure of the laccase molecules.

### 3.6. Phytotoxicity Test

**Figure 10:**
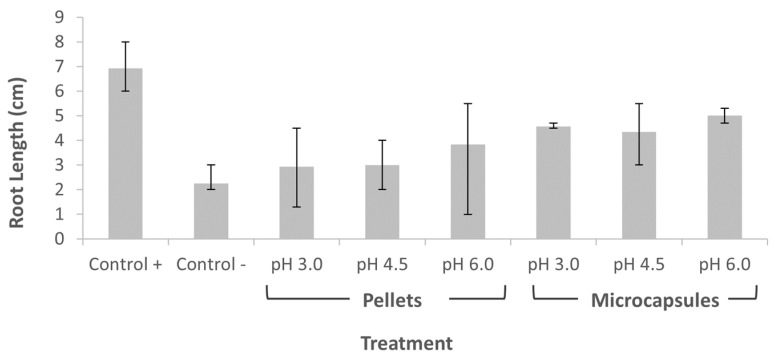
Phytotoxicity test. Milli-Q water and acetaminophen with milli-Q water (1:1 dilution factor) were the positive and negative controls, respectively. Final volume for each replica was 2 mL. Error bars correspond to the absolute error.

Figure 10 shows the phytotoxicity levels obtained for both untreated and treated wastewater samples, after undergoing three operating cycles within the microreactor. Seeds germinated under treated wastewater samples grew significantly more than those exposed to the untreated wastewater samples. According to the ANOVA analysis, the use of laccase immobilized on microcapsules or pellets had a significant impact on toxicity of the treated water compared to the controls (F(3,32) = 40.73 and *p* = 4.0 × 10^−11^). When comparing each immobilization method at different pH values (F(7,16) = 4.479 and *p* = 0.00619), Tukey’s test showed that there was a significant difference between the toxicity of the positive control (Control+) and those reported when using laccase-Al_2_O_3_ pellets. Overall, there was no significant differences in toxicity for the same treatment method; thus, we argue that the pH value has no substantial impact on the toxicity of the solution, but only on the enzyme activity itself. However, the use of the laccase-alginate microcapsules clearly leads to lower phytotoxicity compared with laccase-Al_2_O_3_ pellets. These results demonstrated that the treatments not only provided a route for acetaminophen removal, but also attenuated the toxicity of the components. These results are also in line with the acetaminophen degradation percentages obtained for the treatments involving microcapsules (Figure 7).

## 4. Discussion

The presence of micropollutants in wastewater and freshwater bodies is one of the most significant environmental challenges worldwide [38]. Currently, it is very challenging to remove such pollutants from wastewaters due to their high stability and resistance to physicochemical and biological degradation. Some of the most worrisome micropollutants include pharmaceuticals, personal care products, industrial chemicals, and pesticides [20,39]. Acetaminophen has become one of the most consumed pharmaceutical and personal care products in many countries; it is excreted in both native and metabolized forms to sewer systems, wastewater treatment plants, and natural environments [19]. Biological processes based on enzymes have gained considerable attention over the past decades due to their safeness and reduced undesirable side environmental impacts. However, high concentrations of acetaminophen may inhibit their activity significantly, making them difficult to scale-up and highly unprofitable. In contrast, laccases can carry out degradation processes of pesticides, dyes, pharmaceutical and personal care products while having low energy requirements and mild operational conditions and producing fewer undesirable pollutants after degradation [19,38,39,40]. Some approaches using cell surface-displayed laccase have shown acetaminophen removal efficiency of 70%, in 24 h, Wu, Y., et al. (2019) [39]. This method is based on the expression and localization of laccase on the surface of *Saccharomyces cerevisiae* through engineered biological machinery, allowing the enzyme to be functionally expressed on the cell surface. This surface display laccase biocatalyst could be easily recovered and had high stability after its reuse in a repeated contaminant transformation cyclic operation. However, it is a costly process that requires the maintenance of the cell population; substrate–enzyme interactions suffer from mass transfer limitations. 

Enzyme stability is considerably increased upon encapsulation; polymer entrapment may favor substrate–enzyme interaction [29]. Highly porous materials with high surface areas might facilitate removal of pollutants due to simultaneous adsorption and biocatalytic conversion. In this regard, laccase enzymes have been immobilized on barium alginate for wastewater treatment and for removal of acetaminophen. Authors reported that immobilized laccase showed the maximum removal of 94% in 4 h and that the *K_m_* value for immobilized laccase was lower than that of free laccase, indicating that substrate affinity was probably enhanced by immobilization [40]. Here, the immobilization efficiency was found to be 92.18% and 98.22% for the microcapsules and pellets, respectively, with a maximum acetaminophen removal of 72% and 15% while operating within a continuous flow microreactor with a total retention time of 30 min. We argue that adsorption on Al_2_O_3_ pellets is significantly lower than that obtained with the microcapsules, while the entrapment of the enzyme in an alginate framework favors the enzymatic activity, as the 3D network helps avoiding conformational changes over a wider range of pH and temperature conditions. This increased resilience results in a superior ability to suppress the possible inhibition effects induced by the different components present in real wastewater [38,40].

Our microreactors packed with the laccase-alginate microcapsules allow their intimate contact with the continuous-flow under treatment, therefore, increasing the chances for substrate–enzyme interaction. Microreactors’ volume capacity was 160 μL, while the treated wastewater volume was set to 2 mL, which indicates that the system is capable of treating almost 13 times its maximum capacity per cycle. Here, we take advantage of our microreactors’ high reaction rates and yields, scalability, and more precise control of reaction variables, which results in a significant improvement of the biotransformation performance [41,42]. Moreover, enzyme reusability and an extended enzyme lifetime reduces the operation cost; however, while the laccase-alginate microcapsules can be produced continuously by a microfluidic system, the laccase-Al_2_O_3_ pellets require time-consuming functionalization processes. Moreover, such processes may result in undesirable enzyme crosslinking, leading, in turn, to active site disruption. This was evidenced in the low enzymatic activity measured when quantifying the lost laccase after the treatments.

Here we demonstrated that immobilized laccase can be reused for three operation cycles. This suggests that the wastewater treatment assisted by fluidic microsystems can be scaled up by arranging them in parallel and by replacing the pellets or microcapsules every certain number of working cycles, allowing, thereby, for the treatment of larger volumes of water. This scaling up approach is called numbering-up, as it increases the number of microfluidic devices, instead of incrementing their individual volumetric capacity. This adds up to the low-cost manufacture technique implemented here that might be favorable to increase the profitability in case of eventually scaling-up the process even further [43,44].

Although identifying the resulting chemical specimens after acetaminophen degradation was not in the scope of this study, we tested the phytotoxicity of the untreated and treated wastewater. This is relevant because some by-products resulting from acetaminophen degradation entail an additional challenge associated with mitigating the environmental impacts generated by this pollutant. According to Phong Vo, H., et al. (2019) [19], several transformation processes for acetaminophen have been proposed; more than 20 by-products have been identified during the past 15 years. These metabolites might have different compositions and chemical properties, hence, presenting different levels of toxicity, and could be even more detrimental to living organisms than the parent forms [19,45]. In our case, the difference in seeds growth observed can be related to the acetaminophen degradation and not to pH changes; thus, it can be stated that the by-products present in the treated water are likely to have a lower toxicity than that of the drug itself.

## 5. Conclusions

Emerging micropollutants of high concern such as acetaminophen pose a major remediation challenge due to its recalcitrancy and difficulty to degrade through conventional physicochemical treatment processes. This makes such approaches impractical and extremely costly. Here, we proposed to address this challenge by conducting the treatment in continuous flow microreactors packed with two types of laccase immobilization methods: one was based on the laccase molecules encapsulated in alginate; the other one was based on covalent immobilization on Al_2_O_3_ pellets. For the first case, we used a microfluidic capsule generator to obtain microcapsules with a homogeneous size distribution that was close to that of the Al_2_O_3_ pellets. The immobilization efficiency was found to be 92.18% and 98.22%, while maximum acetaminophen removal achieved was 72% and 15% for the laccase-alginate microcapsules and laccase-Al_2_O_3_ pellets, respectively. We hypothesized that the superior performance of the system packed with microcapsules is related to the longer retention times of acetaminophen molecules within the polymer matrix that leads to more chances for the substrate–enzyme interaction. This contrasts with the very low tortuosity of Al_2_O_3_ pellets and the detrimental conformational changes underwent by laccase molecules during the immobilization process. Reusability of the biocatalysts was tested in three operation cycles, showing a progressive reduction in activity that appeared to be more relevant for the microcapsule-based ones. This correlates with a higher loss of enzyme molecules in such cases. Finally, a phytotoxicity test was conducted to estimate the root growth inhibition potency of treated and untreated wastewater. These results indicate a reduction in phytotoxicity after treatment. Taken together, our findings strongly suggest that the developed biocatalysts, in conjunction with a continuous flow microreactor process scheme, are well suited as an alternative and economically appealing route for the treatment of recalcitrant micropollutants. This is in line with previous reports for microbioreactor systems with immobilized biocatalysts that indicate relatively high biocatalytic activity and pollutant removal efficiency, while maintaining cost-effectiveness because of recycling and reuse of the enzyme [39]. Future work will be dedicated to finding pathways for scaling-up and optimizing this novel process.

## Data Availability

The data and contributions presented in the study are included in the article. Further inquiries can be directed to the corresponding author.
